# Extracapsular Cervical Hematoma After Thyroid Fine-Needle Aspiration Cytology

**DOI:** 10.7759/cureus.29710

**Published:** 2022-09-28

**Authors:** Sara Correia, Diogo Ramalho, Gustavo Rocha, Maria J Oliveira

**Affiliations:** 1 Endocrinology, Centro Hospitalar de Vila Nova de Gaia/Espinho, Vila Nova de Gaia, PRT; 2 Endocrinology and Diabetes, Centro Hospitalar de Vila Nova de Gaia/Espinho, Vila Nova de Gaia, PRT

**Keywords:** intratracheal intubation, thyroid, nodule, aspirative thyroid cytology, cervical hematoma

## Abstract

Fine-needle aspiration cytology (FNAC) is a safe and well-tolerated procedure with high sensitivity and specificity. Serious complications, such as large hematomas, are very rare and should be promptly identified. We present an unusual case of a 71-year-old woman with a massive cervical hematoma that developed near the thyroid capsule after the FNAC of a nodule. CT showed a hematoma measuring 38 x 34 mm, causing deviation of the laryngotracheal axis. The patient was admitted to the intensive care unit for intubation and surgical drainage. This case illustrates that FNAC, despite being considered a safe procedure with infrequent complications, carries the risk of acute life-threatening events that should be taken into account.

## Introduction

Fine needle aspiration cytology (FNAC) is a safe and well-tolerated procedure, with a relevant role in assessing the risk of malignancy of thyroid nodules [1-4]. Most complications have low morbidity and are mostly self-limited [2,4]. Serious complications, such as large hematomas, are very rare and should be quickly identified [2]. 

## Case presentation

A 71-year-old, euthyroid woman was referred for evaluation of a thyroid nodule. She presented with a 19-mm hypoechoic solid oval nodule, with normal vascularization, without microcalcifications, in the lower pole of the left lobe with a score of 4 in the European Thyroid Imaging Reporting and Data System (EU-TIRADS 4). Her medical history was significant for type 2 diabetes mellitus, hypertension, and dyslipidemia. She was chronically medicated with metformin 500 mg, olmesartan 10 mg, and simvastatin 10 mg. The patient denied taking anticoagulants or antiplatelet drugs. No pre-procedure lab testing was performed. She underwent aspiration cytology of the nodule with a 25 G needle and did not comply with the instructions, having swallowed and spoken during the puncture. The procedure was interrupted with no immediate complications. However, a few hours later, she reported sudden cervical swelling with progressive worsening and local pain and denied dyspnea, fever, or stridor.

**Figure 1:**
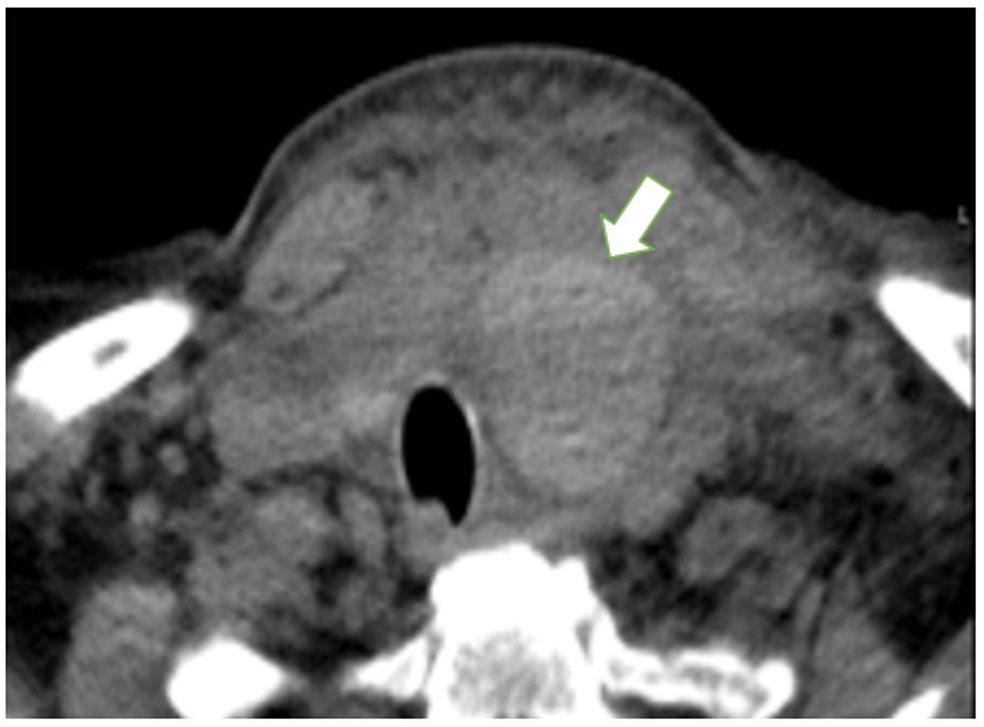
Left latero-cervical hematoma 38 x 34 mm compressing the upper airway

Physical examination revealed edema and stiffness in the anterior cervical region with preserved mobility. There was no subcutaneous emphysema. The oxygen saturation was 100% on room air; her pulse was 90 beats/minute and her blood pressure was 141/69 mm Hg. Hemoglobin, platelets, and coagulation were normal. CT revealed a well-defined hypodense homogeneous mass consistent with a hematoma, adjacent to the left lobe of the thyroid measuring 38 x 34 mm, causing deviation of the laryngotracheal axis contralaterally (Figure 1). 

Angiography of the external carotid artery and the thyrocervical trunk excluded active hemorrhage. The patient was admitted to the intensive care unit with the need for intubation and initiation of anti-edematous measures (hydrocortisone 100 mg every eight hours and furosemide 40 mg every 12 hours); she was started on amoxicillin and clavulanic acid 875 mg/125 mg every 12 hours. After one week of treatment, there was no improvement in her cervical edema or change in the appearance of the hematoma on CT. Thus, surgical drainage of the hematoma was performed. No bleeding points were identified. After surgery, she progressively recovered and there was complete resorption of the hematoma. After extubation on the 13th day, neurological deficits were noticed (right hemiparesis, dysarthria, and dysphonia) and a diagnosis of left lateral frontal cortico-subcortical stroke was made. The hematoma may have caused a restriction in blood flow by compressing the surrounding structures, enough to completely block the blood flow to the cerebral cortex resulting in a stroke. Our stroke unit initiated a rehabilitation program and after discharge, she recovered almost all neurological deficits. Cytology was compatible with a benign follicular nodule (Bethesda category II). 

## Discussion

Post-FNAC serious adverse events seem to be rare, but a systematic record of these does not exist in the literature [2]. A neck hematoma can trigger a deviation of the trachea and can be fatal if it progresses to complete upper airway obstruction [1,2,4]. Even in the absence of dyspnea, endotracheal intubation should be performed as an airway preventive measure [5]. The growth of the hematoma can occur in a few hours or days after the puncture, so immediate detection is not always possible [4]. There are no indications for surgical evaluation of a hematoma, given its rarity. Usually, small to moderate volume hematomas are spontaneously absorbed and do not require hospitalization. If there is impending airway obstruction, intubation and surgical decompression should be considered [1,2]. In the presence of active bleeding or a rapidly developing hematoma, surgery is the first-line treatment [1]. In this case, we initially opted for conservative measures with antibiotics and corticosteroids. The patient was kept under close surveillance and the hematoma was monitored by imaging tests (CT). However, given the unfavorable evolution with its persistence and compressive effect on the trachea, surgical drainage was necessary [4,6].

The presence of factors such as hemorrhagic disorders, anticoagulant/anti-platelet drugs, and hypertension increases the susceptibility to hemorrhagic complications [4,6]. The risk is also increased in the presence of a hypervascularized gland, nodules composed of numerous aberrant vessels with relatively thin walls, and intra-nodular arteriovenous derivations [2,7]. Although the FNAC technique is relatively straightforward and safe, it requires a good amount of experience to master and maintain a skillful puncture technique [3]. The aid of ultrasound-guided puncture supports its execution. The patient's collaboration is also essential through simple attitudes such as not speaking or swallowing during the puncture. If the patient does not comply with these recommendations, the procedure should be stopped immediately. The use of topical lidocaine before FNAC is also recommended by some authors, by reducing pain and facilitating patient collaboration [3]. Beforehand, the doctor must describe the procedure, its possible complications, and the necessary collaboration with the patient [1,2]. Immediately after the procedure, the site must be compressed for a few minutes, followed by the application of ice cold at the puncture site, and observing the patient for at least 30 minutes after the procedure. The patient should be instructed to seek emergency care if edema or persistent acute cervical pain develops [2,3].

## Conclusions

This case intends to illustrate the adverse effects that a relatively simple procedure such as FNAC can have, with complications that, although rare, can be serious. Patient education and collaboration before the procedure are also very important. In the case of a massive cervical hematoma, airway compromise can be fatal, requiring early and appropriate intervention.
